# A *de novo DDX3X* Variant Is Associated With Syndromic Intellectual Disability: Case Report and Literature Review

**DOI:** 10.3389/fped.2020.00303

**Published:** 2020-06-30

**Authors:** Yun Chen, Kai-Yu Liu, Zai-Lan Yang, Xiao-Huan Li, Rui Xu, Hao Zhou

**Affiliations:** ^1^Department of Pediatric Neurology, Guizhou Provincial People's Hospital, Guizhou Medical University, Guiyang, China; ^2^Department of Radiology, Guizhou Provincial People's Hospital, Guizhou Medical University, Guiyang, China

**Keywords:** intellectual disability, exome sequencing, variants, *DDX3X* gene, female

## Abstract

*De novo DDX3X* variants account for 1%–3% of intellectual disability (ID) in females and have been occasionally reported in males. Here, we report a female patient with severe ID and various other features, including epilepsy, movement disorders, behavior problems, sleep disturbance, precocious puberty, dysmorphic features, and hippocampus atrophy. With the use of family-based exome sequencing, we identified a *de novo* pathogenic variant (c.1745dupG/p.S583^*^) in the *DDX3X* gene. However, our patient did not present hypotonia, which is considered a frequent clinical manifestation associated with *DDX3X* variants. While hand stereotypies and sleep disturbance have been occasionally associated with the *DDX3X* spectrum, hippocampus atrophy has not been reported in patients with *DDX3X*-related ID. The investigation further expands the phenotype spectrum for *DDX3X* variants with syndromic intellectual disability, which might help to improve the understanding of *DDX3X*-related intellectual disability or developmental delay.

## Introduction

Intellectual disability (ID) is characterized by serious impairment in intellectual functioning and adaptive behavior (1) and affects ~1%–3% of humans, with a gender bias toward males (1, 2). ID is clinically heterogeneous, accompanied frequently by one or more comorbidities, including seizures, autism spectrum disorder (ASD), and structural and/or functional abnormalities in systems other than the central nervous system (3, 4). Variants in more than 500 candidate genes have been reported in children with intellectual disability, and most of them are associated with neurodevelopment and function of the central nervous system (5, 6). To date, variants of more than 100 genes have been reported to cause monogenic X-linked intellectual disability (XLID) in males (7); however, relatively little is known about X-linked ID genes in females. Recently, an increasing number of X-linked genes, including *PHF6, USP9X* and *DDX3X*, have been identified as carrying *de novo* variants in females with an overlapping but often distinct phenotype in males (8–10).

*DDX3X* is an X-linked gene, encodes a key regulator of the Wnt signaling pathway, and is vital for many cellular processes (11). Recently, *DDX3X* variants have been associated with ID and are considered one of the most common causes of ID in females, accounting for 1%–3% of unexplained ID in females (10). However, patients with ID carrying *DDX3X* variants have been reported in a few studies, and there is no evidence for an obvious correlation between the type of variants and the degree of ID. In the present study, we performed exome sequencing in a proband and her healthy parents to investigate the genetic cause of severe intellectual disability. As a result, we identified a *de novo* frameshift variant in *DDX3X*. Furthermore, we reviewed previously reported patients with *DDX3X* variants and summarized their characteristics, hoping to improve the clinical understanding of the genotype-phenotype correlation.

## Clinical Features and Family History

The patient, 7 years and 5 months of age, who was second-born to healthy non-consanguineous Chinese parents, was referred to the pediatric department due to severe intellectual disability (ID) and epilepsy. She was born at 38 weeks of gestation after an uncomplicated pregnancy and delivery, with a birth weight of 3,100 g (approximately, 50th centile). There were no complications during the perinatal or neonatal periods. Family history is negative for intellectual disability and seizures.

The patient first presented for clinical evaluation at 11 months of age when she was unable to sit without support and did not control her head nor speak any words; the weight and occipito-frontal circumference (OFC) were 8 kg (10th centile) and 44 cm (25th centile), respectively. Her first electroencephalogram (EEG) at 19 months showed increasing theta activity during the awake period but without any seizure. However, at the age of 4 years and 6 months, seizures were captured during video-EEG monitoring and were classified as atypical absence seizures. Subsequently, the patient became seizure free with levetiracetam, and treatment was withdrawn at ~5 years old. However, the patient developed focal seizure with five similar attacks altogether at 7 years and 2 months of age, without motor or language functional regression. Then, she received levetiracetam again after abnormal interictal video-EEG presentations (Supplementary Figure 1, EEG), and was seizure free following daily administration of 5 mg/kg levetiracetam for 3 months.

The patient has shown severe motor and speech delay since infancy, but overall, there has been slow but forward developmental progress. She did not show a social smile until 8 months. Her motor milestones were delayed profoundly, as she developed head control at 12 months, sitting at 18 months, learning to walk at 3 years and 2 months, walking without support at 5 years and running at 6 years slowly with persistent clumsiness. Language acquisition was also obviously delayed but without regression. She said her first words at ~2 years and 4 months, and at 7 years and 5 months, the last visit, she was able to inarticulately express short, three-word sentences. The patient did not present typical autistic-like features, but she was easily agitated and hyperactive, screaming and crying gratuitously, with temper outbursts, intermittent aggressiveness, and exaggerated movements or gestures at times. Moreover, sleep disturbances that consisted of night awaking with screaming spells were observed. However, she did not receive special education services or speech/language therapy.

**Figure 1:**
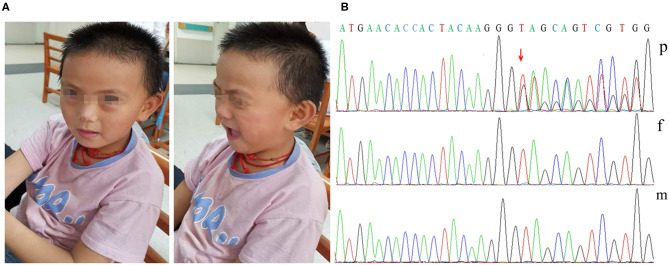
Dysmorphic features and variant analysis. **(A)** Photographs of our patient at 7 years and 5 months showing brachycephaly, a flat face, a thin upper lip, and low-set ears. **(B)** The proband had the c.1745dupG variant of *DDX3X* (p), leading to a premature stop codon; however, her parents had no detectable variants (f and m).

When on admission at 7 years and 5 months, the patient's weight was 25 kg (75th centile), with microcephaly (OFC 49 cm, <3rd centile), dysmorphic features (brachycephaly, a flat face, a thin upper lip, low-set ears; Figure 1A), high-arched palate, wide-based gait, precocious puberty, and mild lower extremity hyperreflexia. Hand stereotypies were frequently observed, presenting as slow fine finger and rapid alternating movements and hand wringing.

**Figure 2:**
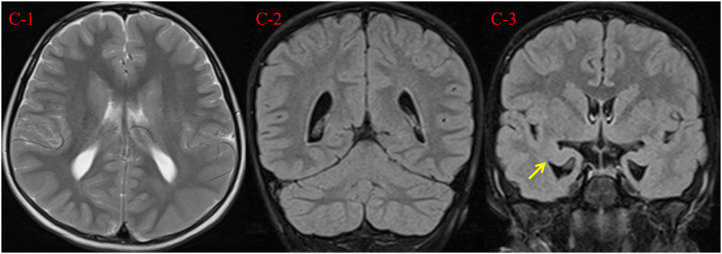
Brain MRI findings at 7 years and 2 months. (C-1, C-2) Axial T2 weighted image and coronal T2 FLAIR demonstrate mild enlargement of the lateral ventricles. (C-3) Coronal T2 FLAIR demonstrates reduced volume of the bilateral hippocampus (arrow), with peculiar temporal horn dilatation.

We performed electrocardiogram (ECG), cardiac ultrasound (CUS), and metabolic testing, including plasma amino acids and acylcarnitines, urinary organic acid analysis, serum creatine kinase, biotinidase activity, folate, homocysteine, copper, blood lactate, and ammonia, all without abnormalities. Her auditory evoked potential was normal. At 7 years and 2 months, testing using the Chinese Revision of the Wechsler Intelligence Scale for Children (WISC)-III showed verbal, performance and full-scale IQ scores of 51, 59, and 51, respectively. Her brain magnetic resonance imaging (MRI) scan at the age of 7 years showed a reduced volume of the bilateral hippocampus, suggesting hippocampus atrophy, with enlargement of the lateral ventricles and peculiar temporal horn dilatation (Figure 2).

## Methodology

### Genetic Findings

Her karyotype analysis was normal. Blood samples were obtained from the patient and her parents for genetic analysis, and informed consent was obtained from her parents. Exome sequencing was performed in the patient and her unaffected parents. Finally, we identified one distinct *de novo* frameshift variant in *DDX3X*: c.1745dupG/p.S583^*^ [ChrX (GRCh37), NM_001193416.1; Figure 1B]; no likely pathogenic copy number variant was identified through an analysis of the exome data. The variant identified in our patient has not been previously reported in the literature or in any public database. The variant pathogenicity was then determined according to the American College of Medical Genetics and Genomics (ACMG) guidelines (12): This variant showed very strong evidence of pathogenicity since it was a null variant (variant leading to a premature stop codon) in *DDX3X* (PVS1), was a *de novo* variant (PS2), was absent in population databases (PM2), and further met the supporting criteria of PP4. In conclusion, we regarded c.1745dupG/p.S583^*^ as a pathogenic variant (PVS1+PS2+PM2+PP4).

### Literature Search

To characterize *DDX3X* variants in intellectual disability and to explore the likely association between the phenotype and *DDX3X* gene variants, we performed a systematic literature search in the PubMed database, the Human Gene Mutation Database (HGMD), the Online Mendelian Inheritance in Man (OMIM) and the China National Knowledge Infrastructure (CNKI) databases for publications using “intellectual disability” or “developmental delay” and “*DDX3X*” as the keywords (until January 9th, 2020). In addition to our patient, we identified 96 patients with *DDX3X* variants reported previously (10, 13–19). We excluded four patients reported in a single publication and one fetus from further analysis since all comprehensive clinical presentations were unavailable (17, 18). Complete nomenclature of variants was derived from the Mutalyzer (https://mutalyzer.nl/name-checker). Finally, we summarized the clinical and genetic characteristics of the remaining 91 patients reported in 10 publications (10, 13–16, 18–22); the proportion of females was ~89% (81/91), and only 10 male patients have been described. The findings are presented in Table 1 and Supplementary Table 1.

**Table 1:** Clinical features of affected females and males with *DDX3X* variants.

		**F**									**M**			**Total (%)**
**Reference**		**(10)**	**(18)**	**(19)**	**(16)**	**(14)**	**(20)**	**(21)**	**(22)**	**Present case**	**(10)**	**(15)**	**(13)**	
ID/DD		38/38	28/28	3/3	2/2	2/2	1/1	6/6	1/1	**+**	5/5	3/3	2/2	92/92 (100.0)
Growth	Microcephaly	12/38	7/28	2/3	0/2	2/2	0/1	1/6	0/1	**+**	2/5	0/3	0/2	27/92 (29.3)
	Low weight	12/38	3/13	0/3	0/2	0/2	NA	1/6	1/1	**–**	NA	1/3	NA	18/69 (26.1)
	Macrocephaly	0/38	0/28	0/3	0/2	0/2	0/1	0/6	0/1	**–**	0/5	0/3	2/2	2/92 (2.2)
Craniofacial Abnormalities	Dysmorphic features	30/38	19/28	3/3	2/2	2/2	1/1	6/6	1/1	**+**	2/5	3/3	0/2	70/92 (76.1)
	Plagiocephaly	0/38	1/13	1/3	0/2	0/2	0/1	0/6	0/1	**–**	0/5	2/3	0/2	4/77 (5.2)
	Brachycephaly	0/38	0/13	0/3	1/2	0/2	0/1	1/6	0/1	**+**	3/5	1/3	0/2	7/77 (9.1)
Neurology	Hypotonia	29/38	19/28	2/3	1/2	2/2	0/1	0/6	0/1	**–**	0/5	2/3	0/2	55/92 (59.8)
	Movement disorder	17/38	17/28	3/3	1/2	2/2	0/1	2/6	0/1	**+**	3/5	3/3	2/2	51/92 (55.4)
	Behavior problems	20/38	6/28	0/3	0/2	0/2	0/1	2/6	1/1	**+**	1/5	0/3	1/2	32/92 (34.8)
	Epilepsy	6/38	1/13	1/3	0/2	2/2	0/1	1/6	0/1	**+**	0/5	1/3	0/2	13/77 (16.9)
Brain MRI	Abnormal	13/37	18/20	3/3	0/2	2/2	0/1	1/4	1/1	**1/1**	NA	2/3	2/2	52/76 (68.4)
	CCH	13/37	NA	3/3	NA	2/2	0/1	1/4	1/1	**–**	NA	1/3	0/2	
	VE	13/37	NA	3/3	NA	2/2	0/1	0/4	1/1	**+**	NA	0/3	2/2	
	CM	4/37	NA	2/3	NA	1/2	0/1	0/4	0/1	**–**	NA	0/3	0/2	
	CCA	0/37	NA	0/3	NA	0/2	0/1	0/4	0/1	**–**	NA	0/3	2/2	
	HA	0/37	NA	0/3	NA	0/2	0/1	0/4	0/1	**+**	NA	0/3	0/2	
	Other^*^	0/37	NA	0/3	NA	0/2	1/1	0/4	0/1	**–**	NA	2/3	0/2	
Sleep disturbance		0/38	0/28	0/3	0/2	0/2	NA	2/6	0/1	**+**	NA	0/3	0/2	3/86 (3.5)
Ophthalmological abnormalities		13/38	9/28	2/3	1/2	1/2	0/1	3/6	1/1	**–**	2/5	2/3	0/2	34/92 (37.0)
Skin abnormalities		14/38	5/28	NA	1/2	0/2	NA	2/6	1/1	**–**	0/5	0/3	0/2	23/88 (26.1)
Hyperlaxity		14/38	2/13	0/3	0/2	0/2	NA	0/6	1/1	**–**	0/5	0/3	0/2	17/76 (22.4)
Scoliosis		4/38	0/28	2/3	0/2	2/2	NA	2/6	1/1	**–**	0/5	0/3	0/2	11/91 (12.1)
CHD		NA	5/7	NA	NA	2/2	NA	1/6	NA	**–**	NA	3/3	NA	11/18 (61.1)
Dyspnea		NA	5/28	NA	0/2	2/2	NA	0/6	0/1	**–**	NA	1/3	0/2	8/45 (17.8)
Audiological abnormalities		3/38	0/28	1/3	0/2	2/2	1/1	0/6	0/1	**–**	0/5	1/3	0/2	8/92 (8.7)
Precocious puberty		5/38	NA	NA	NA	0/2	NA	0/3	0/1	**+**	NA	0/3	0/2	6/50 (12.0)

CCA, corpus callosum atrophy; CCH, corpus callosum hypoplasia; CHD, congenital heart disease; CM, cortical malformation; DD, developmental delay; F, female; HA, hippocampus atrophy; ID, intellectual disability; M, male; NA, not available; Other

* * including watershed infarcts, Dandy–Walker variant and arachnoid cyst; VE, ventricular enlargement*.

## Discussion

**Figure 3:**
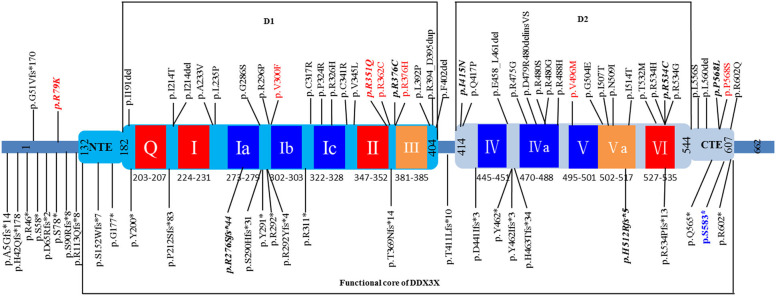
Schematic view of the DDX3X protein structure based on Song et al. (26) showing D1D2, NTE, and CTE fragments. The 12 highly conserved sequence motifs are color coded: red, ATP binding; blue, RNA binding; orange, coordination between ATP and RNA binding. Unique variants (total: 73) are listed in the schematic view, showing the location of the variant identified in our patient (p.S583* in blue). The variants found in affected males are shown in red, and recurrent variants are indicated with bold italics. The 8 splice site variants identified in published cohorts are not shown in this figure.

In this study, we performed trio-exome sequencing for a female patient with severe intellectual disability (ID) and identified a novel *de novo* pathogenic variant (c.1745dupG/p.S583^*^) of *DDX3X*. *DDX3X* encodes a 662-amino-acid protein and contains a helicase core composed of two RecA-like domains (D1D2) and N- and C-terminal extensions (NTE and CTE, respectively) beyond the two RecA-like domains. D1D2 harbors 12 highly conserved sequence motifs involved in nucleoside triphosphate binding, RNA binding, helicase activity and hydrolysis (23). Both the N- and C-terminal domains are involved in the specific biological role of the protein (24, 25). Therefore, the functional core of DDX3X was recently redefined to contain the NTE-D1D2-CTE fragment [residues 132-607; Figure 3; (23, 26)].

DDX3X has been associated with many cellular processes, such as cell cycle control, apoptosis, and tumorigenesis (27, 28). In 2014, the Deciphering Developmental Disorders Study identified *de novo* variants of *DDX3X* in 4 female patients with ID and proposed *DDX3X* as a candidate ID gene (17). In a large cohort of 6,072 patients with unexplained ID (2,659 females), *de novo* variants of *DDX3X* were identified in 38 females (10). According to previous reports, *de novo* variants of *DDX3X* account for 1%-3% of unexplained ID cases in females (10, 18).

To date, a total of 80 unique *DDX3X* variants have been described as causing intellectual disability or developmental delay, including 36 missense variants, 18 frameshift variants, 11 nonsense variants, eight splice site variants and seven small in-frame deletion/duplication variants, and most of the variants are located within the NTE-D1D2-CTE fragment of DDX3X (Figure 3 and Supplementary Table 1). *De novo* patterns occurred in all female patients, and two of the *de novo* variants (c.573_575delCAT/p.I191del and c.1805G>A/p.R602Q) are mosaic in the proband, with allele fractions of 21% and 14%, respectively (18). Eight unique variants were identified in 10 male patients, including three *de novo* variants (15, 18), four maternally inherited variants (10, 13, 18), and one variant with unknown parental status (c.1702C>T/p.P568S) (15). The pathogenicity of these maternally inherited variants was determined by functional testing, which suggested partial loss of function of DDX3X as the most likely pathogenic mechanism (10, 13). Pathogenicity of the variant with unknown parental status was predicted to be damaging on the basis of similar phenotypes, family history and *in silico* prediction algorithms, including the consensus predictor, PredictSNP2 (15). Moreover, eight recurrent variants were reported, including c.236G>A/p.R79K, c.828_831delAGAG/p.R276Sfs^*^44, c.1052G>A/p.R351Q, c.1126C>T/p.R376C, c.1244T>A/p.I415N, c.1535_1536delAT/p.H512Rfs^*^5,c.1600C>T/p.R534C, and c.1703C>T/p.P568L (Supplementary Table 2).

The frequent clinical presentations reported include ID and/or developmental delay (DD) (92/92), dysmorphic facial features (70/92), hypotonia (55/92), movement disorders (51/92, e.g., dyskinesia, spasticity, stiff-legged or wide-based gait, ataxia, and choreoathetosis), structural brain abnormalities (52/76), ophthalmological abnormalities (34/92), behavior problems (32/92, e.g., agitation and hyperactivity, aggression and autism spectrum disorder), and microcephaly (27/92). The most frequent structural brain abnormalities reported include corpus callosum hypoplasia and ventricular enlargement. Thirteen patients experienced seizures, and five patients individually presented with general akinetic seizures, absence seizures, tonic-clonic seizures, spasms and febrile seizures; however, other clinical data, including EEG and treatment, were not available [Supplementary Table 3; (10, 14, 15, 18, 19, 21)]. Though all reported female and male patients had ID and/or DD and a majority suffered from movement disorders, compared to female patients, male patients rarely showed hypotonia, skin abnormalities, hyperlaxity, or precocious puberty, and often showed brachycephaly and plagiocephaly.

There is substantial clinical overlap between the phenotype in our patient and those of the females described to date, including severe ID, microcephaly, movement disorder, behavior problems, epilepsy, dysmorphic features, and ventricular enlargement. However, our patient also presented features that are distinct from those of the patients reported previously. In comparison to published data, our patient did not present hypotonia, which was a common feature in previous reports. Furthermore, hand stereotypies and temporal horn dilatation have been occasionally associated with the *DDX3X* spectrum (15, 19), while sleep disturbance has been reported in only two female patients (21), and hippocampus atrophy has not been reported in patients with *DDX3X*-related ID to date.

There is no evidence for an obvious correlation between the location of variants and the severity of phenotype in previous reports (10, 18). However, variants located in the D1D2 and CTE fragments seemed more likely to be associated with moderate-severe or severe intellectual disability in our literature review (Supplementary Table 1). In addition, review of clinical data revealed that the same recurrent variants may result in very similar phenotypes (Supplementary Table 2). For example, two of three patients with the *de novo* c.1703C>T/p.P568L variant exhibited hypotonia, microcephaly, visual problems, scoliosis, seizures, ventricular enlargement, and corpus callosum hypoplasia; two patients with the c.236G>A/p.R79K variant had progressive spastic paraparesis, tremor, behavioral problems, decreased lower extremity strength, ventricular enlargement and corpus callosum hypoplasia. Conversely, the two patients with c.828_831delAGAG/p.R276Sfs^*^44 had no abnormal neurological or brain imaging findings, corresponding with only mild to moderate ID.

To assess the correlation between the type of variants and the severity of clinical phenotype, we investigated the frequency of severe ID in patients who carry missense and frameshift or nonsense variants [predicted loss of function (LoF)]. These missense and LoF variants were found in 81.3% (65/80) of the cohort, and most of these variants fall within the NTE-D1D2-CTE fragments. Surprisingly, patients with missense variants (45.2%, 19/42) were more likely to have severe ID than patients with frameshift or nonsense variants (13.0%, 3/23) (*p* < 0.01). We consider these LoF variants likely to undergo nonsense-mediated RNA decay (NMD), which may have different effects on phenotype than missense variants. NMD is thought to serve as an mRNA surveillance mechanism to prevent the synthesis of truncated proteins that would have the potential to have toxic effects, such as dominant negative interactions (29, 30). The association between severe ID and the type of *DDX3X* variants likely suggests the possibility of a dominant negative impact of *DDX3X* missense variants in severely affected individuals; in contrast, patients with frameshift or nonsense LoF variants may show milder phenotypes because of the NMD mechanism, which decreases the levels of potentially deleterious proteins (30). However, the definite phenotype-genotype correlations need to be studied with a larger, more fully phenotyped patient cohort in the future.

Somatic variants of the *DDX3X* gene have also been identified in a variety of malignancies (19). In two studies, five variants (c.1600C>T/p.R534C, c.1703C>T/p.P568L, c.641T>C/p.I214T, c.931C>T/p.R311^*^, c.1084C>T/p.R362C) were reported to occur somatically in association with malignant melanoma, medulloblastoma, and esophageal squamous cell carcinoma (10, 18). However, malignancy was not reported in any patients in a previous study except one patient diagnosed with global DD harboring a *DDX3X* missense variant (c.1511G>A/p.G504E) (19). A larger study needs to be performed to elucidate whether patients with ID/DD harboring *DDX3X* germline variants are at greater risk of developing malignancies.

## Conclusion

We identified a novel pathogenic nonsense variant of *DDX3X* in a female patient suffering from severe ID and various other features. This is the first study to report a Chinese case of ID with a *DDX3X* variant. According to our systematic literature review, most of the variants are located within the two RecA-like domains of *DDX3X*. Patients with missense variants were more likely to have severe ID compared to the patients with a frameshift or nonsense variants. This investigation further expanded the number of *DDX3X* variants and their associated phenotypic spectrum, which might help to improve the understanding of *DDX3X*-related intellectual disability or developmental delay. However, the phenotype-genotype correlations need to be studied with a larger, fully phenotyped patient cohort in the future.

## Data Availability Statement

All datasets generated for this study are included in the article/Supplementary Material.

## Ethics Statement

The study was performed in accordance with the Declaration of Helsinki of the World Medical Association (WMA), and written informed consent was obtained from the parents for the research study, presentation of photographs, clinical details, and publication of this case report.

## Author Contributions

YC drafted the manuscript. YC, K-YL, Z-LY, X-HL, and RX contributed to the clinical data acquisition. YC and HZ contributed to the analysis and genetic evaluation. HZ critically revised the manuscript.

## Conflict of Interest

The authors declare that the research was conducted in the absence of any commercial or financial relationships that could be construed as a potential conflict of interest.
