# Supplementary material for: A de novo DDX3X Variant Is Associated With Syndromic Intellectual Disability: Case Report and Literature Review
Source: Front Pediatr. 2020 Jun 30;8:303. doi: 10.3389/fped.2020.00303 (PMC7344189; doi:10.3389/fped.2020.00303)
Supplement: Supplementary file 1 [file Data_Sheet_1.pdf]

Supplementary Table 1 Amino acid changes and the phenotype are listed for each identified variant

Patient	Gender	Age (years)	Nucleotide change	Amino acid change	Inheritance pattern	Amino acid position (CSM)	ID/DD	Reference
1	F	NA	c.14_17delCAGT	p.A5Gfs*14	de novo		DD and/or ID	(18)
2	F	12	c.46-2A>C	p.?	de novo		mild-moderate	(10)
3	F	6	c.46-2A>G	p.?	de novo		moderate disability	(10)
4	F	NA	c.126_129delTTTA	p.H42Qfs*178	de novo		DD and/or ID	(18)
5	F	8	c.136C>T	p.R46*	de novo		moderate disability	(10)
6	F	11	c.147delT	p.G51Vfs*170	de novo		DD	(21)
7	F	NA	c.173C>A	p.S58*	de novo		DD and/or ID	(18)
8	F	1.5	c.192dupA	p.D65Rfs*2	de novo		DD	(18)
9	F	8	c.233C>G	p.S78*	de novo		mild-moderate	(10)
10	M	29	c.236G>A	p.R79K	maternal		mild-moderate	(13)
11	M	25	c.236G>A	p.R79K	maternal		mild-moderate	(13)
12	F	33	c.269dupG	p.S90Rfs*8	de novo		mild-moderate	(10)
13	F	2	c.284+1G>A	p.?	de novo		ID	(18)
14	F	4.5	c.336dupC	p.R113Qfs*8	de novo		ID	(18)
15	M	NA	c.443+3A>T	p.?	de novo		DD and/or ID	(18)
16	F	6	c.453_454delTT	p.S152Wfs*7	de novo	NTE	DD	(18)
17	F	NA	c.529G>T	p.G177*	de novo	NTE	mild-moderate	(16)
18	F	NA	c.573_575delCAT	p.I191del	De novo, mosaic 21%	D1	DD and/or ID	(18)
19	F	1	c.599dupA	p.Y200*	de novo	D1	DD	(10)
20	F	4	c.632dupT	p.P212Sfs*83	de novo	D1	DD	(21)
21	F	10	c.641_643delTCA	p.I214del	de novo	D1	severe disability	(19)
22	F	2	c.641T>C	p.I214T	de novo	D1	mild-moderate	(10)
23	F	15	c.698C>T	p.A233V	de novo	D1	moderate-severe	(10)
24	F	7	c.704T>C	p.L235P	de novo	D1	severe disability	(10)
25	F	17	c.766-1G>C	p.?	de novo		severe disability	(10)
26	F	15	c.828_831delAGAG	p.R276Sfs*44	de novo	D1 (Ia)	moderate disability	(21)
27	F	12	c.828_831delAGAG	p.R276Sfs*44	de novo	D1 (Ia)	mild disability	(21)
28	F	NA	c.856G>A	p.G286S	No paternal sample	D1	severe disability	(16)
29	F	5	c.865-1G>A	p.?	de novo		DD	(18)
30	F	2	c.865-2A>G	p.?	de novo		mild-moderate	(10)
31	F	8	c.868delT	p.S290Hfs*31	de novo	D1	moderate disability	(10)
32	F	13	c.873C>A	p.Y291*	de novo	D1	moderate disability	(10)
33	F	NA	c.873_874insTATA	p.R292Yfs*4	de novo	D1	DD and/or ID	(18)
34	F	NA	c.874C>T	p.R292*	de novo	D1	DD and/or ID	(18)
35	F	NA	c.887G>C	p.R296P	de novo	D1	DD and/or ID	(18)
36	M	5	c.898G>T	p.V300F	maternal	D1	severe disability	(10)
37	F	10	c.931C>T	p.R311*	de novo	D1	severe disability	(10)
38	F	NA	c.949T>C	p.C317R	de novo	D1	DD and/or ID	(18)
39	F	NA	c.971C>G	p.P324R	de novo	D1 (Ic)	DD and/or ID	(18)

Patient	Gender	Age (years)	Nucleotide change	Amino acid change	Inheritance pattern	Amino acid position (CSM)	ID/DD	Reference
40	F	13	c.977G>A	p.R326H	de novo	D1(Ic)	severe disability	(10)
41	F	0.5	c.1021T>C	p.C341R	de novo	D1	DD	(18)
42	F	NA	c.1033G>C	p.V345L	de novo	D1	DD and/or ID	(18)
43	M	24	c.1052G>A	p.R351Q	maternal	D1(II)	moderate-severe	(10)
44	M	NA	c.1052G>A	p.R351Q	maternal	D1(II)	DD and/or ID	(18)
45	M	36	c.1084C>T	p.R362C	maternal	D1	mild disability	(10)
46	F	3	c.1105dupA	p.T369Nfs*14	de novo	D1	mild-moderate	(10)
47	F	4	c.1126C>T	p.R376C	de novo	D1	severe disability	(10)
48	F	8	c.1126C>T	p.R376C	de novo	D1	severe disability	(10)
49	F	3	c.1126C>T	p.R376C	de novo	D1	moderate disability	(10)
50	M	7	c.1127G>A	p.R376H	de novo	D1	moderate-severe	(15)
51	F	2.6	c.1170+1dupG	p.?	de novo		moderate-severe	(21)
52	F	2	c.1175T>C	p.L392P	de novo	D1	severe disability	(10)
53	F	NA	c.1180_1185dupCGTGAT	p.R394_D395dup	de novo	D1	DD and/or ID	(18)
54	F	14	c.1206_1208delCTT	p.F402del	de novo	D1	severe disability	(18)
55	F	9	c.1229_1230dupCT	p.T411Lfs*10	de novo		moderate disability	(10)
56	F	NA	c.1244T>A	p.I415N	de novo	D2	DD	(18)
57	F	NA	c.1244T>A	p.I415N	de novo	D2	DD and/or ID	(18)
58	F	9	c.1250A>C	p.Q417P	de novo	D2	severe disability	(10)
59	F	2	c.1321delG	p.D441Ifs*3	de novo	D2	DD	(10)
60	F	3	c.1371_1382del GGAGGATTTCTT	p.E458_L461del	de novo	D2	moderate-severe DD	(21)
61	F	4	c.1383dupA	p.Y462Ifs*3	de novo	D2	moderate disability	(10)
62	F	NA	c.1386C>G	p.Y462*	no parental samples	D2	DD and/or ID	(18)
63	F	5	c.1384_1385dupTA	p.H463Tfs*34	de novo	D2	moderate disability	(10)
64	F	3	c.1423C>G	p.R475G	de novo	D2(IVa)	moderate-severe	(10)
65	F	2	c.1436_1439delinsTCTC	p.D479R480delinsVS	de novo	D2(IVa)	severe disability	(19)
66	F	4.5	c.1438A>G	p.R480G	de novo	D2(IVa)	severe disability	(18)
67	F	14	c.1440A>T	p.R480S	de novo	D2(IVa)	severe disability	(10)
68	F	18	c.1463G>A	p.R488H	de novo	D2(IVa)	severe disability	(10)
69	M	8	c.1486G>A	p.V496M	de novo	D2(V)	moderate disability	(15)
70	F	11	c.1511G>A	p.G504E	de novo	D2(Va)	severe disability	(19)
71	F	3	c.1520T>C	p.I507T	de novo	D2(Va)	severe disability	(10)
72	F	7	c.1526A>T	p.N509I	de novo	D2(Va)	severe disability	(10)
73	F	18	c.1535_1536delAT	p.H512Rfs*5	de novo	D2(Va)	moderate disability	(10)
74	F	10	c.1535_1536delAT	p.H512Rfs*5	de novo	D2(Va)	mild disability	(10)
75	F	10	c.1535_1536delAT	p.H512Rfs*5	de novo	D2(Va)	ID/DD	(22)
76	F	1	c.1541T>C	p.I514T	de novo	D2(Va)	DD	(10)
77	F	NA	c.1595C>T	p.T532M	de novo	D2(VI)	DD and/or ID	(18)
78	F	47	c.1600C>T	p.R534C	de novo	D2(VI)	DD	(18)
79	F	1	c.1600C>T	p.R534C	de novo	D2(VI)	DD	(18)

Patient	Gender	Age (years)	Nucleotide change	Amino acid change	Inheritance pattern	Amino acid position (CSM)	ID/DD	Reference
80	F	4	c.1600C>G	p.R534G	de novo	D2(VI)	severe disability	(14)
81	F	18	c.1600dupC	p.R534Pfs*13	de novo	D2(VI)	mild disability	(10)
82	F	13	c.1601G>A	p.R534H	de novo	D2(VI)	severe disability	(10)
83	F	NA	c.1667T>C	p.L556S	de novo	CTE	ID	(20)
84	F	7	c.1678_1680delCTT	p.L560del	de novo	CTE	mild disability	(10)
85	F	9	c.1693C>T	p.Q565*	de novo	CTE	severe disability	(10)
86	M	16	c.1702C>T	p.P568S	no parental samples	CTE	severe disability	(15)
87	F	11	c.1703C>T	p.P568L	de novo	CTE	severe disability	(10)
88	F	10	c.1703C>T	p.P568L	de novo	CTE	severe disability	(14)
89	F	7	c.1703C>T	p.P568L	de novo	CTE	DD	(18)
90	F	NA	c.1804C>T	p.R602*	de novo	CTE	DD and/or ID	(18)
91	F	NA	c.1805G>A	p.R602Q	de novo, mosaic 14%	CTE	DD and/or ID	(18)
92	F	7	c.1745dupG	p.S583*	de novo	CTE	severe disability	Present case

F: Female; M: Male; DD: Developmental Delay; ID: Intellectual Disability; DD and/or ID: Further information is unavailable; NA: Not Available; CSM: Conserved Sequence Motifs
