# Supplementary material for: A de novo DDX3X Variant Is Associated With Syndromic Intellectual Disability: Case Report and Literature Review
Source: Front Pediatr. 2020 Jun 30;8:303. doi: 10.3389/fped.2020.00303 (PMC7344189; doi:10.3389/fped.2020.00303)
Supplement: Supplementary file 2 [file Data_Sheet_2.pdf]

Supplementary Table 2 Clinical features of patients with recurrent *DDX3X* variants

Variant position	Variant type	AA position (CSM)	Years/ sex	Seizure type	ID/ DD	Other neurologic findings	Non-neurologic findings	Brain MRI findings	Ref.
c.236G>A/ p.R79K	De novo		29/M	NS	ID	Progressive spastic paraparesis, behavior problems, decreased lower extremity strength	Macrocephaly, dysarthria	CCH, VE	(13)
c.236G>A/ p.R79K	De novo		25/M	NS	ID	Progressive spastic paraparesis, tremor, behavior problems, decreased lower extremity strength	Learning disability, mixed expressive-receptive language disorder	CCH, VE	(13)
c.828_831delAGAG/p.R276Sfs*44	De novo	D1 (Ia)	15/F	NS	Moderate	Normal	Dysmorphic features	NP	(21)
c.828_831delAGAG/p.R276Sfs*44	De novo	D1 (Ia)	12/F	NS	Mild	Normal	Dysmorphic features	NP	(21)
c.1126C>T/ p.R376C	De novo	D1	4/F	NS	Severe	Hypotonia, movement disorder	Hyperlaxity, visual problems	CCH	(10)
c.1126C>T/ p.R376C	De novo	D1	8/F	NS	Severe	Movement disorder, behavior problems	Hyperlaxity, skin abnormalities, precocious puberty	Normal	(10)
c.1126C>T/ p.R376C	De novo	D1	3/M	NS	Mild	Hypotonia, behavior problems	Normal	CCH, VE	(10)
c.1535_1536delAT/p.H512Rfs*5	De novo	D2(Va)	18/F	NS	Moderate	Normal	Hyperlaxity, precocious puberity	Normal	(10)
c.1535_1536delAT/p.H512Rfs*5	De novo	D2(Va)	10/F	NS	Mild	Hypotonia	Normal	VE	(10)
c.1535_1536delAT/p.H512Rfs*5	De novo	D2(Va)	10/F	NS	ID	Hypotonia, ADHD	Microcephaly, dysmorphic features	CCH, VE	(22)
c.1600C>T/ p.R534C	De novo	D2(VI)	47/F	NS	Severe	Hypotonia	Microcephaly, short stature, dysmorphic features, unilateral renal agenesis, difficulty feeding as a child	CCH, polymicrogyria	(18)
c.1600C>T/ p.R534C	De novo	D2(VI)	1/F	GTCS	DD	Dystonia and choreoathetoid movements	IUGR, poor feeding, low weight, frequent apnea episodes, central blindness	Abnormal signal in the thalamus	(18)
c.1703C>T/ p.P568L	De novo	CTE	11/F	NA	Severe	Hypotonia, movement disorder	Microcephaly, visual problems, scoliosis, low weight	CCH, VE	(10)
c.1703C>T/ p.P568L	De novo	CTE	10/F	IS	Severe	Hypotonia	Microcephaly, short stature, hypermobility, visual problems, hearing loss, dysmorphic features, scoliosis, respiratory distress, VSD	CCH, VE, delayed myelination	(14)
c.1703C>T/ p.P568L	De novo	CTE	7/F	NS	ID/ DD	Hypotonia with decreased muscle mass	Microcephaly, short stature, cortical visual impairment, dysmorphic features	VE, delayed myelination	(18)

AA: Amino Acid; ADHD: Attention Deficit Hyperactivity Disorder; AS: Absence Seizures; ASD: Atrial Septal Defect; CCH: Corpus Callosum Hypoplasia; CM: Cortical Malformation; CSM: Conserved Sequence Motifs; CTE: C-terminal extensions of DDX3X; D1/D2: The functional core of DDX3X, composed

of two RecA-like domains; DD: Developmental Delay; F: Female; GTCS: Generalized Tonic-Clonic Seizures; ID: Intellectual Disability; IS: Infantile Spasms; IUGR: Intrauterine Growth Retardation; M: Male; MRI: Magnetic Resonance Imaging; NA: Not Available; NP: Not Performed; NS: No Seizures; PDA: Patent Ductus Arteriosus; Ref.: Reference; VE: Ventricular Enlargement; VSD: Ventricular Septal Defect. Variants c.1244T>A/p.I415N and c.1052G>A/p.R351Q were not listed as comprehensive clinical presentations were unavailable.
