# Supplementary material for: A de novo DDX3X Variant Is Associated With Syndromic Intellectual Disability: Case Report and Literature Review
Source: Front Pediatr. 2020 Jun 30;8:303. doi: 10.3389/fped.2020.00303 (PMC7344189; doi:10.3389/fped.2020.00303)
Supplement: Supplementary file 3 [file Data_Sheet_3.pdf]

Supplementary Table 3 Clinical features of *DDX3X*-related patients accompanied with epilepsy

Variant position	Variant type	AA position (CSM)	Years /sex	Seizure type	ID/DD	Other neurologic findings	Nonneurologic findings	Brain MRI findings	Ref.
c.641_643del TCA/ p.I214del	De novo	D1	10/F	Akinetic seizure	Severe	Hypotonia, spasticity, dystonia, strabismus, stereotypies	IUGR, dysmorphic features, microcephaly, severe scoliosis, hearing loss	Polymicrogyria, CCH, VE, temporal poles hypoplasia	(19)
c.873C>A/ p.Y291*	De novo	D1	13/F	NA	Moderate	Hypotonia, movement disorder, behavior problems	Skin abnormalities, hyperlaxity, microcephaly	NP	(10)
c.931C>T/ p.R311*	De novo	D1	10/F	NA	Severe	Hypotonia, behavior problems	Skin abnormalities, hyperlaxity, precocious puberty	Normal	(10)
c.1127G>A/ p.R376H	De novo	D1	7/M	AS	Severe	Hypotonia, repetitive movements	Plagiocephaly, dysmorphic features, myopia, astigmatism, recurrent otitis media, ASD, PDA	Watershed infarcts	(15)
c.1321delG/ p.D441Ifs*3	De novo	D2	2/F	NA	DD	Hypotonia, behavior problems	Visual problems, low weight	Normal	(10)
c.1371_1382del GGAGGATTTCT/ p.E458_L461del	De novo	D2	3/F	NA	Moderate-severe DD	Hypertonia, stiff legs, dystonic episodes	Reduced subcutaneous fat, slender fingers, talipes valgus deformity, scoliosis, disordered sleep	CCH	(21)
c.1440A>T/ p.R480S	De novo	D2(IVa)	14/F	NA	Severe	Hypotonia, movement disorder, behavior problems	Skin abnormalities, visual problem, low weight	Normal	(10)
c.1520T>C/ p.I507T	De novo	D2(Va)	3/F	NA	Severe	Hypotonia, movement disorder	Skin abnormalities, visual problem, hearing loss	CCH, CM, VE	(10)
c.1600C>G/ p.R534G	De novo	D2(VI)	4/F	NA	Severe	Hypotonia	IUGR, microcephaly, short stature, hypermobility, visual problems, hearing loss, dysmorphic features, ASD, PDA, scoliosis	CCH, VE, CM, delayed myelination	(14)
c.1600C>T/ p.R534C	De novo	D2(VI)	1/F	GTCS	DD	Dystonia and choreoathetoid movements	IUGR, poor feeding, low weight, frequent apnea episodes, problems with body temperature control, central blindness	Abnormal signal in the thalamus	(18)
c.1703C>T/ p.P568L	De novo	CTE	11/F	NA	Severe	Hypotonia, movement disorder	Visual problems, scoliosis, low weight, microcephaly	CCH, VE	(10)
c.1703C>T/ p.P568L	De novo	CTE	10/F	IS	Severe	Hypotonia	Microcephaly, short stature, hypermobility, visual problems, hearing loss, dysmorphic features, scoliosis, respiratory distress, VSD	CCH, VE, delayed myelination	(14)
c.1745dupG/ p.S583*	De novo	CTE	7/F	AAS, FS	Severe	Hand stereotypies, behavior problems, wide-based gait, mild lower extremity hyperreflexia	Dysmorphic features, microcephaly, brachycephaly, precocious puberty, sleep disturbance	HA, peculiar temporal horn dilatation, VE	Present case

AA: Amino Acid; AAS: Atypical Absence Seizures; AS: Absence Seizures; ASD: Atrial Septal Defect; CCH: Corpus Callosum Hypoplasia; CM: Cortical Malformation; CSM: Conserved Sequence Motifs; CTE: C-terminal extensions of DDX3X; D1/D2: The functional core of DDX3X, composed of two RecA-like domains; DD: Developmental Delay; F: Female; FS: Febrile Seizures; GTCS: Generalized Tonic-Clonic Seizures; HA: Hippocampus Atrophy; ID: Intellectual Disability; IS: Infantile Spasms; IUGR: Intrauterine Growth Retardation; M: Male; MRI: Magnetic Resonance Imaging; NA: Not Available; NP: Not Performed; PDA: Patent Ductus Arteriosus; Ref.: Reference; VE: Ventricular Enlargement; VSD: Ventricular Septal Defect
