# Supplementary material for: A de novo DDX3X Variant Is Associated With Syndromic Intellectual Disability: Case Report and Literature Review
Source: Front Pediatr. 2020 Jun 30;8:303. doi: 10.3389/fped.2020.00303 (PMC7344189; doi:10.3389/fped.2020.00303)
Supplement: Supplementary file 4 [file Data_Sheet_4.PDF]

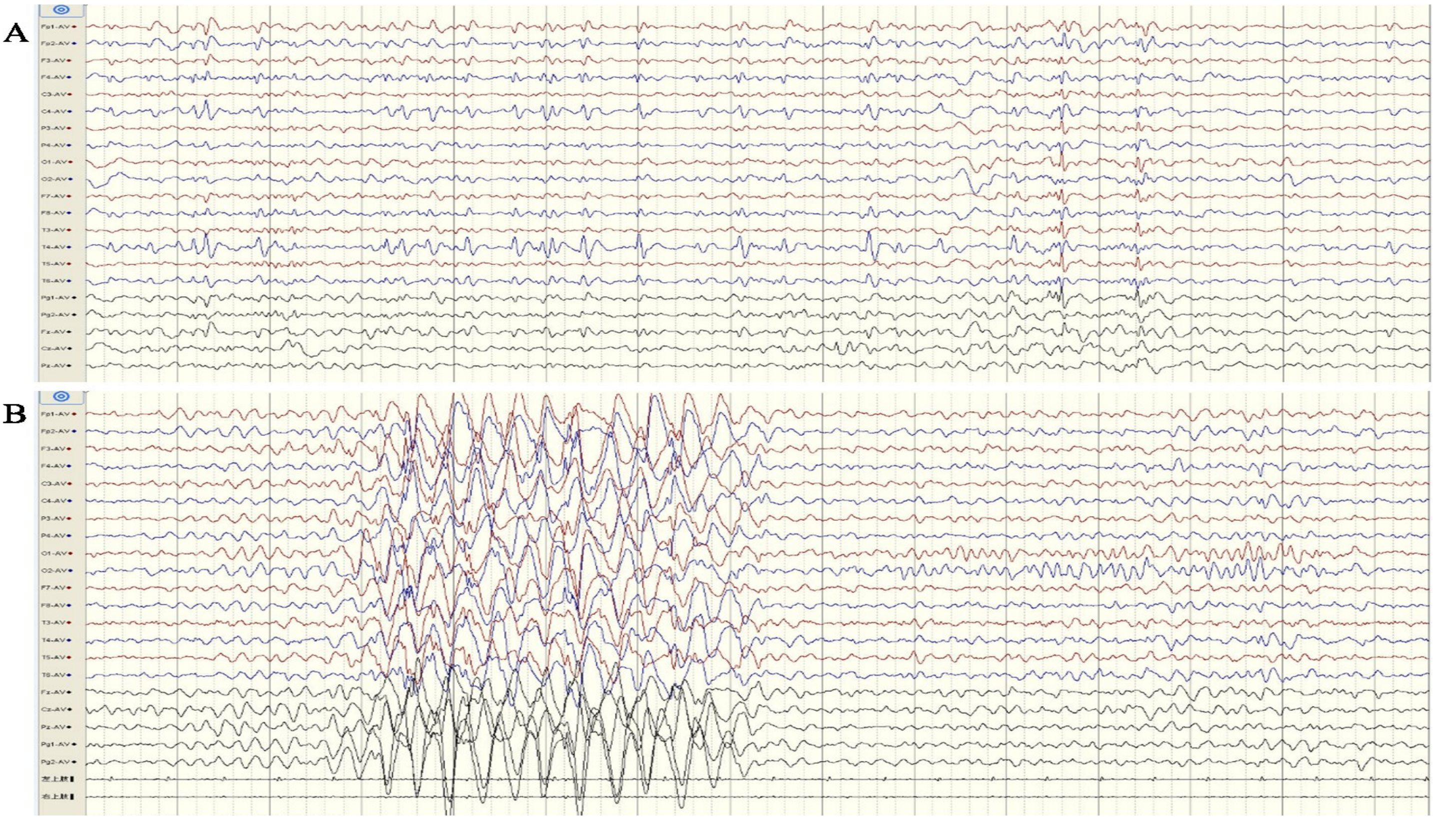

Representative EEG tracings from our patient with a de novo variant of *DDX3X*. **A** shows a slow background rhythm with focal epileptiform discharges that are more prominent in the right temporal and central regions, with occasional secondary generalization, and **B** shows atypical absence seizures with EEG showing generalized 2.5-3.5 Hz moderate-extreme high amplitude slow waves at 7 years and 2 months.
